# A Comparative Study of the Effects of Platinum (II) Complexes on β-Amyloid Aggregation: Potential Neurodrug Applications

**DOI:** 10.3390/ijms22063015

**Published:** 2021-03-16

**Authors:** Sara La Manna, Daniele Florio, Ilaria Iacobucci, Fabiana Napolitano, Ilaria De Benedictis, Anna Maria Malfitano, Maria Monti, Mauro Ravera, Elisabetta Gabano, Daniela Marasco

**Affiliations:** 1 Department of Pharmacy, University of Naples “Federico II”, 80131 Naples, Italy; sara.lamanna@unina.it (S.L.M.); floriodaniele1@gmail.com (D.F.); ilariadebenedictis2308@gmail.com (I.D.B.); 2 Department of Chemical Sciences, CEINGE Biotecnologie Avanzate S.c.a r.l., “University of Naples Federico II”, 80131 Naples, Italy; ilaria.iacobucci@unina.it (I.I.); montimar@unina.it (M.M.); 3 Department of Translational Medical Science, University of Naples “Federico II”, 80131 Naples, Italy; fabiananapolitano94@gmail.com (F.N.); annamaria.malfitano@unina.it (A.M.M.); 4 Department of Sciences and Technological Innovation (DiSIT), University of Piemonte Orientale “A. Avogadro”, 15121 Alessandria, Italy; mauro.ravera@uniupo.it (M.R.); elisabetta.gabano@uniupo.it (E.G.)

**Keywords:** amyloid aggregation, metallodrugs, platinum (II) compounds, anti-aggregation properties

## Abstract

Herein the effects of three platinum complexes, namely (*SP*-4-2)-(2,2′-bipyridine)dichloridoplatinum(II), Pt-bpy, (*SP*-4-2)-dichlorido(1,10-phenanthroline) platinum(II), Pt-phen, and (*SP*-4-2)-chlorido(2,2′:6′,2′′-terpyridine)platinum(II) chloride, Pt-terpy, on the aggregation of an amyloid model system derived from the C-terminal domain of Aβ peptide (Aβ_21–40_) were investigated. Thioflavin T (ThT) binding assays revealed the ability of Pt(II) compounds to repress amyloid aggregation in a dose-dependent way, whereas the ability of Aβ_21–40_ peptide to interfere with ligand field of metal complexes was analyzed through UV-Vis absorption spectroscopy and electrospray ionization mass spectrometry. Spectroscopic data provided micromolar EC_50_ values and allowed to assess that the observed inhibition of amyloid aggregation is due to the formation of adducts between Aβ_21–40_ peptide and complexes upon the release of labile ligands as chloride and that they can explore different modes of coordination toward Aβ_21–40_ with respect to the entire Aβ_1–40_ polypeptide. In addition, conformational studies through circular dichroism (CD) spectroscopy suggested that Pt-terpy induces soluble β-structures of monomeric Aβ_21–40_, thus limiting self-recognition. Noticeably, Pt-terpy demonstrated the ability to reduce the cytotoxicity of amyloid peptide in human SH-SY5Y neuroblastoma cells. Presented data corroborate the hypothesis to enlarge the application field of already known metal-based agents to neurodegenerative diseases, as potential neurodrugs.

## 1. Introduction

**Figure 1:**
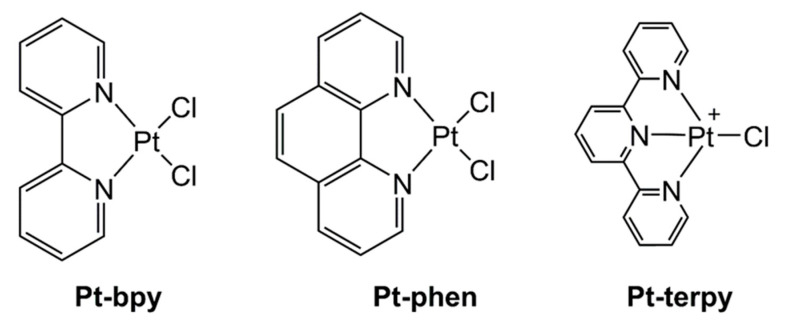
Structure of the Pt(II) complexes investigated in the present work (bpy = 2,2′-bipyridine, phen = 1,10-phenanthroline, terpy = 2,2′:6′,2′′-terpyridine).

Aβ-amyloid peptides (1–40 and 1–42) form oligo- and multi-meric aggregates that evolve into fibrils recovered in the brains of patients affected by Alzheimer’s disease (AD) [1]. Peptide sequences at early stages of aggregation, both as monomers and oligomers, are largely disordered and able to create coordination bonds with transition metal ions, that change the kinetics and mechanism of self-recognition [2]. Transition metal complexes are able to modulate/inhibit amyloids’ aggregation and possibly their toxicity through different mechanisms [3]: (i) coordination chemistry, (ii) oxidative and (iii) proteolytic reactions [4] for peptide modifications. These processes depend on tunable features of complexes, including the oxidation state of the ion and the coordination geometry of the metal center [5,6]. In addition, β-amyloid peptides are involved in the homeostasis of several bio-active metal ions as copper(I, II) and zinc(II). Several studies outlined how the Aβ peptide/Cu interactions are mediated by the N-terminal region of Aβ-amyloid involving His^6^, His^13^, and His^14^ and that these interactions modulate the aggregation and toxicity profiles of the entire Aβ_1–40_ sequence [7]. In general, Pt(II) and Ru(III) [8] complexes are more stable and redox inert with respect to Zn-, Cu-, or Fe-based compounds, thus, they can have major chances of success to prevent toxic effects of Aβ oligomerization in a coordinative mechanism and to serve as potential neurodrugs [9]. Many investigations reported on the ability of Pt(II) complexes to interfere with amyloid aggregation. Initial studies involved Pt(II) compounds bearing 1,10-phenanthroline (phen)-based bidentate ligands along with two monodentate ligands (e.g., chlorides), as an inhibitor of aggregation of Aβ_1–40_ via the exchange of the latter ligands followed by the coordination to the imidazoles of His residues of the N-terminal region of Aβ_1–40_ [10]. π–π interactions between the phen moiety and aromatic Phe^4^, Tyr^10^ and Phe^19^ aid the formation of the [Pt^II^(phen)–(Aβ)]^2+^ adduct that suppressed aggregation and limited the neurotoxicity of Aβ in mouse hippocampal tissue [10]. Other studies reported on the interaction of Pt-phen complexes not only with the entire Aβ_1–40_ sequence but also with smaller regions of the N-terminal domain [11]: NMR, X-ray absorption spectroscopy (XAS), mass spectrometry (MS), and molecular modeling investigations [12] further confirmed that the planar hydrophobic phen ligand stabilized histidine-protein adducts [13]. In detail, the non-covalent interactions of phen with the protein, and the coordination to the Pt(II) center of two His, limited the coordination of Cu(II) and Zn(II) [14,15]. Similarly, other Pt-complexes containing planar hydrophobic ligands demonstrated the ability to interact with the first 28 residues of Aβ-amyloid, Aβ_1–28,_ exhibiting different binding modes between the Pt(II) center and the polypeptide chain [16,17]. An important application is related to the detection of amyloid fibrillation through the employment of self-assembled luminescent Pt(II) complexes [18,19]. Very recently, we carried out several studies focusing on the effects of several metallo-drugs on the aggregation of several peptide sequences assumed as amyloid models [20,21,22,23,24,25]. The fragment spanning 21–40 residues of the C-terminal domain of Aβ_1–40_ (Aβ_21–40_, Table 1) was tested with Pd(II)-, Pt(II)- and Au(III) compounds featuring 2-(2′-pyridyl)benzimidazole [26], Pt(II) complexes with β-hydroxy dithiocinnamic esters [9] and Ru(II)-based CO-releasing molecules featuring bidentate benzimidazole and terpyridine derivatives [27]. They all revealed able to modulate self-assembly of different amyloid sequences employing coordination or oxidative mechanisms. Herein we investigated the interaction between the almost unexplored C-terminal fragment Aβ_21–40,_ with a small series of Pt(II) compounds that were already demonstrated the ability to bind to the entire β-amyloid polypeptide [6]. The selected complexes were: (*SP*-4-2)-(2,2′-ridine)dichloridoplatinum(II), Pt-bpy, (*SP*-4-2)-dichlorido(1,10-phenanthroline) platinum(II), Pt-phen, and (*SP*-4-2)-chlorido(2,2′:6′,′′-terpyridine)platinum(II) chloride, Pt-terpy) (Figure 1). Moreover, in order to assess the specificity of the observed results, we also investigated the potential effects of the Pt(II) complexes on a mutated version of Aβ_21–40_, namely mutAβ_21–40_ (Table 1), bearing the point mutation G^37^/D that was reported to suppress amyloid aggregation of the entire Aβ_1–42_ [28].

## 2. Results

### 2.1. Effects of Pt Complexes on Thioflavin T (ThT) Aassay of Aβ_21–40_

**Figure 2:**
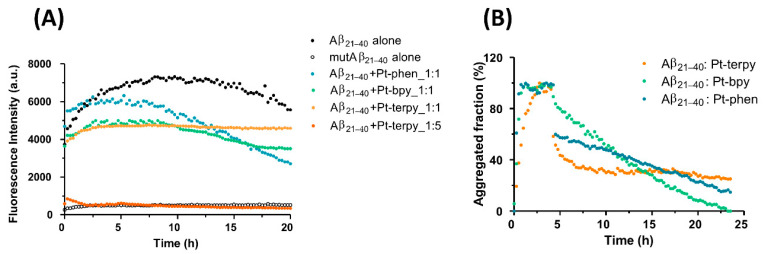
Time course of ThT fluorescence emission intensity of Aβ_21–40_ peptides alone and upon the addition of Pt-phen, Pt-bpy, and Pt-terpy at (**A**) t = 0, at the indicated molar ratios, and (**B**) after 5 h, time of addition is indicated with an arrow, at 1:1 ratio.

The ability of Pt complexes to affect the aggregation process of Aβ_21–40_ was monitored through the analysis, over time, of Thioflavin T (ThT) fluorescence emission, reported in Figure 2A. Two molar ratios for peptide: metal complexes were possible for Pt-terpy (i.e., 1:1 and 1:5), whereas Pt-phen and Pt-bpy resulted not soluble at the 1:5 ratio ([Pt] = 500 µM). The starting value of fluorescence different from 0 is ascribable to an immediate partial oligomerization during sample preparation, that however requires more time (~5 h) to reach saturated ThT signal, as already observed [9,29]. Over time, Aβ_21–40_ alone reached a saturated signal after 6 h of stirring, while mutAβ_21–40_ is not able to aggregate for 20 h, as expected. At 1:1 ratio, Aβ_21–40_: Pt complexes, the effects of metal compounds are clearly suppressive of the self-assembly process even at initial times of aggregation. This is more evident for Pt-terpy and Pt-bpy complexes, while almost negligible for Pt-phen. A complete inhibition of the aggregation is displayed by 1:5 ratio of Pt-terpy that provides a signal similar to mutAβ_21–40_, for the entire duration of the analysis.

In addition, we investigated if Pt(II)-complexes were able not only to inhibit Aβ_21–40_ aggregation but also to modulate its disaggregation. Hence the ThT-intensity profiles versus time upon the addition of complexes at 1:1 ratio, on pre-formed soluble aggregates are reported in Figure 2B. Interestingly, we observed a decrease of ThT fluorescence intensity upon the addition of all three complexes with different times of decrease. Once again, Pt-terpy proved to be the most effective since it caused an immediate decrease of aggregated fractions greater than 60% while for Pt-phen the time of decrease was similar, but a reduction of the fraction was of ~40%. Conversely, the disaggregation caused by Pt-bpy was slower and only after 15 h provided effects comparable to those exhibited by the other two metal complexes.

### 2.2. Analysis of the Adducts between Aβ_21–40_ and Pt(II) Complexes through Mass Spectrometry

**Figure 3:**
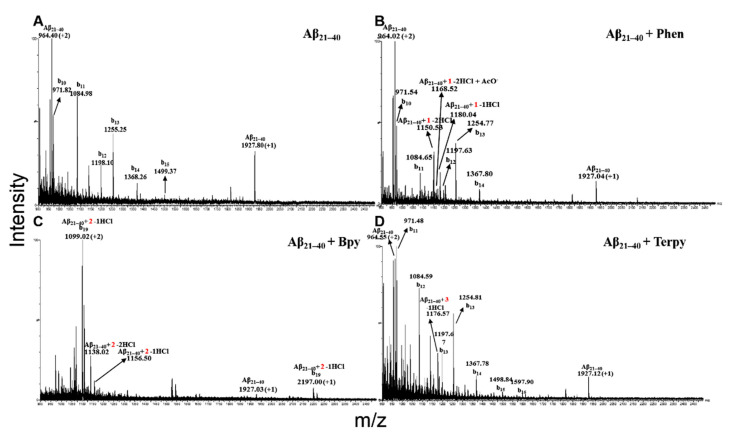
ESI-MS spectra of Aβ_21–40_ peptide at 24 h of incubation with Pt-derivates. (**A**) Isolated Aβ_21–40_. (**B**) Aβ_21–40_ + Pt-phen. (**C**) Aβ_21–40_ + Pt-bpy. (**D**) Aβ_21–40_ + Pt-terpy. The presence of the Pt moieties in the ions is indicated with 1, 2 and 3, respectively. Fragmentation ions are also indicated.

Samples of Aβ_21–40_ alone and incubated with the Pt compounds were analyzed at two different times (0 and 24 h) by electrospray ionization mass spectrometry (ESI-MS) [30]. As reported in Figure 3, ESI-MS spectra recorded at 24 h, showed that all the three Pt complexes are able to bind one chain of the Aβ_21–40_, and all the species detected at the longest time were already present at t = 0 (Table 2). By a detailed inspection of the MS spectra of Aβ_21–40_ with Pt-phen and Pt-bpy (Figure 3B–C), two double-charged ions were present: one at higher *m*/*z* values (Pt-phen *m*/*z* = 1168.52 Da; Pt-bpy *m*/*z* = 1156.50 Da) and a second peak at lower *m*/*z* values (Pt-phen *m*/*z* = 1150.52 Da; Pt-bpy *m*/*z* = 1138.02 Da), deriving by the loss of one and two chloride ions, respectively. These latter peaks increased over time, as already observed in similar studies [27]. In addition, the species missing two chloride ions are flanked by the presence of an adduct deriving from the substitution of a chloride with an acetate ion, present in the buffer (Pt-phen *m*/*z* = 1180.04 Da; Pt-bpy *m*/*z* = 1168.05 Da). Aβ_21–40_ formed an adduct with one molecule of Pt-terpy (Figure 3D), losing the only chloride present, as demonstrated by the presence of the double charged ion at *m*/*z* 1176.57 Da. Moreover, in the ESI-MS acquiring conditions, the isolated Aβ_21–40_ peptide provided an in-source fragmentation phenomena, generating b series fragments (Figure 3A), as previously reported [26]. The fragmentation of free Aβ_21–40_ peptide persisted also in the presence of Pt-phen (Figure 3B) and Pt-terpy (Figure 3D), since a large amount of peptide was unbound. Conversely, in the presence of Pt-bpy no free peptide fragmentation was detectable (Figure 3C), since the largest part of Aβ_21–40_ was bound to the Pt-compound. However, differently from other complexes, Aβ_21–40_/Pt-bpy is the unique adduct to provide fragmentation, as confirmed by mono- and double-charged species of b_20_ C-terminal fragment evidenced in the ESI-MS spectrum. As a control experiment, mutAβ_21–40_ alone and in presence of Pt-terpy was also studied, and no peaks attributable to the adduct formed by the peptide and the metal complex were evidenced (see Figure S1, Supplementary Material). MS data demonstrated that all Pt-compounds bind Aβ_21–40_ with 1:1 stoichiometry and that the adduct with Pt-bpy resulted more abundant with respect to the others, as demonstrated by the dominant species in the spectrum associated with the complex and its b_19_ fragment.

### 2.3. Changes of Absorption Spectral Features of Pt(II)-Complexes Caused by Aβ_21–40_

**Figure 4:**
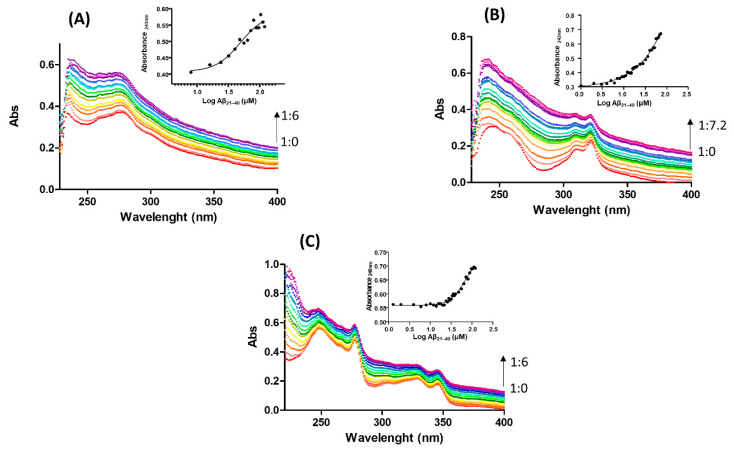
Absorption spectra of (**A**) Pt-phen, (**B**) Pt-bpy, (**C**) Pt-terpy upon the addition of increasing amount of Aβ_2–40_. Arrow indicates ratio variation between complex and peptide. As insets UV intensities at indicated wavelengths versus Log concentration of Aβ_21–40_ are reported.

UV/Vis absorption spectroscopy was employed to detect potential variations of the ligand fields of investigated Pt(II) complexes introduced by the presence of amyloid peptide. The spectra of the Pt(II) complexes in the 200–400 nm region are characterized by the presence of more or less resolved or evident absorptions, which are combinations of internal π→π* transitions of the ligand and metal-to-ligand charge transfer transitions [31,32,33]. As reported in Figure 4, the enhancement of absorbances upon increasing amounts of Aβ_21–40_ for all three investigated Pt(II) complexes clearly suggests a substitution of ligands around metal ion from coordinating residues deriving from peptide side chains.

For Pt-phen and Pt-terpy (Figure 4A,C), the titration with Aβ_21–40_ reached saturated values, providing an estimation of EC_50_ (half-maximal effective concentration) of 49 ± 20 µM (inset of Figure 4A) for Pt-phen and 68 ± 14 µM for Pt-terpy (inset of Figure 4C), respectively. On the contrary, Pt-bpy did not provide saturation even if a greater ratio (7.2:1, peptide: complex) was reached with respect to the other two complexes (6:1). To ensure that the increase of absorbance is not due to a dilution effect, similar assays, at the same concentration of the metal complexes were carried out by adding the same volumes of solvent as in the titration. As shown in Figure S2, slight intensity variations go in the opposite direction (decrease of signal) upon dilution, that cannot interfere with observed results of titration.

### 2.4. Soluble Pt-Terpy Complex Stabilizes the β-Structure of Aβ_21–40_

**Figure 5:**
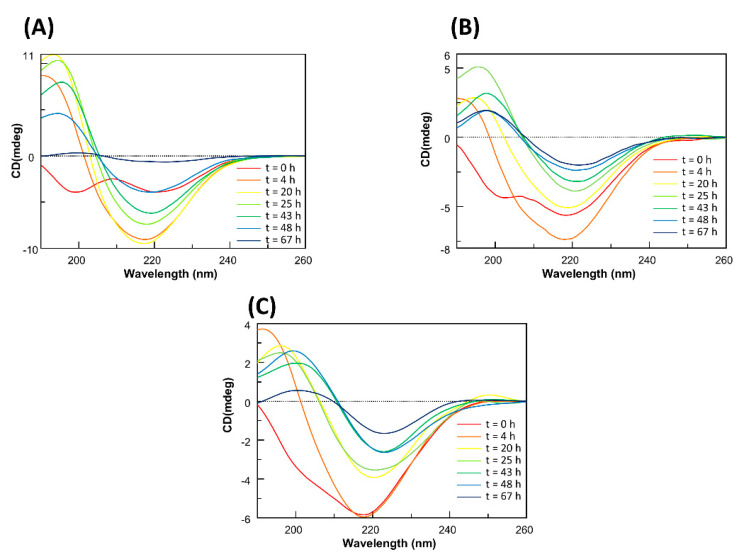
Overlay of CD spectra of Aβ_21–40_ (**A**) alone, incubated with Pt-terpy at (**B**) 1:1 and (**C**) 1:5 peptide: Pt(II) compound molar ratio.

To gain insights into the potential effects of Pt-complexes on the Aβ_21–40_ conformation, circular dichroism (CD) investigations were carried out. Often the poor water solubility of metal-complexes requires handling with only freshly prepared samples greatly hampering the long time of analysis, in our case, since Pt-terpy revealed as the most potent inhibitor of ThT fluorescence and resulted soluble in aqueous solution, samples containing Aβ_21–40_/Pt-terpy adducts were analyzed in long interval times (up to 3 d). The overlays of CD spectra of Aβ_21–40_ alone and Aβ_21–40_ + Pt-terpy at 1:1 and 1:5 ratios are reported in Figure 5. For Aβ_21–40_, the CD spectrum of a freshly prepared sample suggests the co-existence of random and β-structures (Figure 5A, red line), as confirmed by the deconvolution of the spectra (see Table S1 Supplementary Material) where their percentages are 23.4 and 55 %, respectively. Deconvolution at t = 0 estimates secondary structure contents in completely soluble conditions. In the following 20 h (yellow line) the conformation evolves toward a more defined β-structure, that remains prevalent, even if, from this time, a progressive reduction of Cotton effect is observable due to an aggregation/precipitation phenomenon. This behavior persists till 67 h when CD signal is almost null (blue line). For Aβ_21–40_ + Pt-terpy at 1:1 ratio, the t = 0 h CD profile (Figure 5B, red line) suggest a stabilization of β-structure with respect to that observed in absence of Pt complex (34.1% for β and 44.9% for random. Table S1), as already reported for similar complexes to Aβ_21–40_ [9] and the coordination of Zn(II) to Aβ_1–40_ [34]. This conformation seems to be reinforced after 4 h (orange line) and maintained in the following hours. After 20 h (yellow line) the decrease of Cotton effect is downsized with respect to Aβ_21–40_ alone since even at 67 h CD signal is meaningful (blue line). A similar behavior was exhibited by Aβ_21–40_ + Pt-terpy at 1:5 ratio (Figure 5C): in this case, the stabilization of β-structure by the Pt complex, at early stages of analysis, is more evident with respect to 1:1 sample, since the spectrum at t = 0 h (red line) is almost superimposable to that at t = 4 h (orange line).

As control experiments, the time course of the CD with mutAβ_21–40_ peptide in presence and absence of Pt-terpy was evaluated. Differently from the profile of the wild-type sequence, in the overlay of CD spectra of the mutant peptide (Figure S3A) the absence of conformational transition through β-structures for all time of analysis, confirmed the inability to form amyloid assemblies by these sequences, corroborating ThT assay results. Nevertheless, the reduction of Cotton effect starting from ~40 h is likely due to an amorphous aggregation propensity of the entire sequence. Similarly to wild-type sequence, the addition of Pt(II) terpy provided a stabilization of β-structure (Figure S3B).

### 2.5. Soluble Pt-Terpy Complex Inhibits Cytotoxic Effects of Aβ_21–40_ Peptide in SH-SY5Y Cells

**Figure 6:**
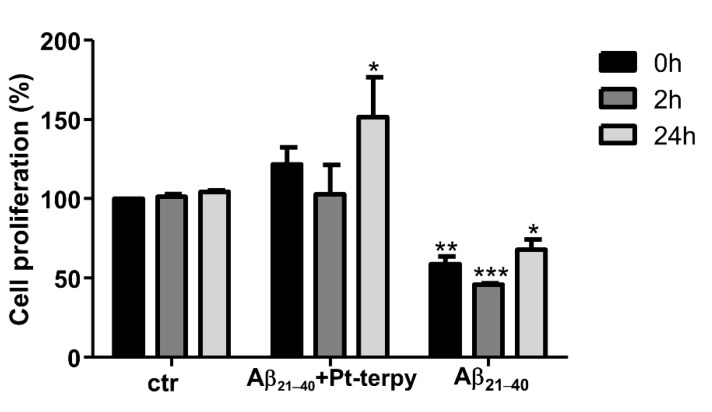
Cell proliferation assays in SH-SY5Y cells treated with Aβ_21__–40_ alone and Aβ_21__–40_: Pt-terpy at 1:5 peptide to metal compounds molar ratio at three different times. Statistical analysis was calculated by GraphPad Prism 7 by two-way Anova with Dunnett’s multiple comparison test (* *p* ≤ 0.05, ** *p* ≤ 0.005 and *** *p* ≤ 0.0005 vs. basal (at corresponding time)).

The ability of the Pt-terpy complex to reduce the toxicity of Aβ_21–40_ was assessed using human SH-SY5Y neuroblastoma cells. Cell proliferation was evaluated after treating cells with the peptide alone or in the presence of Pt-terpy, at 1:5 ratio peptide: complex (Figure 6) that represents the limit of water solubility for metal compound. Pt-terpy demonstrated the efficiency to interfere with the aggregation of Aβ_21–40_ (Figure 2). With respect to cells alone (that present, as expected, comparable proliferation), Aβ_21–40_ peptide caused a significant reduction of cell proliferation even at t = 0 of analysis: this can be likely due to both partial aggregated state of prepared sample (as also observed for starting values of ThT analysis, Figure 2A) and to a long time of cellular analysis. However, the highest reduction (~40%) was exhibited at 2 h while, after 24 h, it demonstrated limited effects on proliferation that increased at ~75%. This behavior was already observed for similar amyloid systems in the transition from initial toxic small and soluble aggregates toward later, larger, and less toxic assemblies [35]. On the other hand, the adduct Aβ_21–40_ + Pt-terpy exhibited a quite different behavior, indeed a rescue of cell proliferation with respect to Aβ_21– 40_ was observed that clearly indicates the inhibition of Aβ_21–40_ toxic effects [36].

## 3. Discussion

Metal-based compounds present exclusive mechanisms of action (MOAs) that are finely regulated by properties exerted by different (i) kinetics, (ii) coordination geometries, (iii) redox states (iv) and hydrophobicity of ligands [6]. These features could provide unexplored therapeutic approaches employing metal complexes as drugs [11]. Our recent investigations outlined the versatility of metal-based anticancer drugs to be applied in other fields of drug discovery as neurodegenerative diseases: in them, short peptide sequences demonstrated suited model systems of grater related proteins to investigated different MOAs of Pt-, Ru- and Au- complexes as effectors of amyloid aggregation [9,26,27]. Herein the modulation of self-assembly of the C-terminal region of β-amyloid, Aβ_21–40,_ by several Pt(II) derivatives, containing different ligands, was investigated. Time-course analysis of ThT fluorescence indicates a clear reduction of the aggregative process mostly for Pt-terpy and Pt-bpy complexes, a 1:1 ratio. Noticeably this reduction translates both in inhibitory and disaggregating effects on pre-formed soluble oligomers. ESI-MS spectrometry and UV/Vis absorption spectroscopy experiments indicate that the modulation of amyloid aggregation depends on the formation of adducts between metal-compounds and the Aβ_21–40_ peptide, a 1:1 stoichiometry. By comparing present and previous data [9,26] Aβ_21–40_ fragment was very sensitive to the action of Pt(II)-complexes and in this study, the coordination of side chains of amino acids of the peptide to the Pt(II) ion occurs upon the release of the two or one coordinated Cl ions. Another important result of the present study relies on the druggability of the fragment 21–40 of β-amyloid: indeed, the same metal complexes investigated on full-length or N-terminal regions of Aβ_1–40/42_ sequences, revealed able to target a C-terminal region of amyloid polypeptide, even if with a different MOA excluding the His-coordination to Pt(II) ion. Interestingly, the presence of Pt-terpy was able to inhibit the Aβ_21–40_ toxicity in human SH-SY5Y neuroblastoma cells restoring cell proliferation. This is an important indication of the inhibition of Aβ_21–40_ toxic effects on living cells and not in simpler model systems. In conclusion, we have presented several promising results of anti-aggregation properties of Pt(II) complexes, employing a coordinative mechanism, and their potential therapeutic application in neurodegenerative diseases.

## 4. Materials and Methods

### 4.1. Peptide Synthesis

Wild-type and mutAβ_21–40_ peptides were synthesized as already reported [9,26,27]. The sequences are reported in Table 1, after purification they were lyophilized and treated with HFIP to ensure a monomeric state and then stored at −20 °C until use.

### 4.2. Synthesis of the Complexes

Pt-terpy was obtained by using the traditional reaction between (*SP*-4-2)-dichlorido(cycloocta-1,5-diene)platinum(II), [PtCl_2_(cod)], and 2,2′:6′,2′′-terpyridine [37]. Pt-bpy was synthesized by a microwave-assisted reaction between K_2_[PtCl_4_] and 2,2′-bipyridine [38]. Pt-phen was synthesized adapting the same procedure employed for Pt-bpy. The chemicals used for the synthesis were obtained from common commercial sources, used as received and without further purification. Microwave irradiation was performed by using a CEM Discover^®^ SP System equipped (CEM corporation, England) with a focused single-mode and self-tuning cavity, an air-cooling system, and an automated power control based on temperature feedback, supplying power in 1 W increments from 0 to 300 W. The purity of all the compounds was assessed by elemental analysis on an EA3000 CHN Elemental Analyzer (EuroVector, Milano, Italy). Electrospray ionization mass spectra (ESI-MS) were obtained using a Waters HPLC-MS instrument equipped with a 3100 mass detector (Agilent, Milan, Italy), setting the source and desolvation temperatures at 150 °C and 250 °C, respectively, and using nitrogen both as a drying and as a nebulizing gas. The cone and the capillary voltages were usually 30 V or 20 V and 2.70 kV, respectively. Quasi-molecular ion peaks [M + H]^+^ or [M + Na]^+^ were assigned on the basis of the *m*/*z* values and of the simulated isotope distribution patterns. The NMR spectra were measured on an NMR-Bruker Avance III spectrometer (Bruker, Milan, Italy) operating at 500 MHz (^1^H), 125.7 MHz (^13^C), and 107.2 MHz (^195^Pt with a spectral window of 2000 ppm), respectively. ^1^H and ^13^C NMR chemical shifts were reported in parts per million (ppm) referenced to solvent resonances. ^195^Pt NMR spectra were recorded using a solution of K_2_PtCl_4_ in saturated aqueous KCl as the external reference. The shift for K_2_PtCl_4_ was adjusted to −1628 ppm from Na_2_PtCl_6_ (δ = 0 ppm).

#### Synthesis of (SP-4-2)-dichlorido(1,10-phenanthroline)platinum(II), Pt-phen

In a 10-mL microwave vessel to a solution of K_2_[PtCl_4_] (30 mg) in water (3 mL), 1 eq of 1,10-phenantroline in ethanol (1 mL) was added. After capping it, the vessel was introduced into the microwave oven and heated to 60 °C over a 5-min ramp period and then kept at this temperature for 15 min under stirring (power 10 W). After cooling it to room temperature, the vessel was removed from the cavity and further cooled to 0 °C to increase the amount of yellow precipitate, which was then separated by centrifugation and washed with water, methanol, and diethyl ether. Yield: 51%. ESI-MS (positive ion mode): found 469 *m*/*z*; calcd. for C_12_H_8_Cl_2_N_2_NaPt [M + Na]^+^ 469 *m*/*z*. ^1^H-NMR (DMF-d_7_) δ: 8.26 (dd, 2H, H3 and H8, *J*^3^ = 8.2 Hz, *J*^3^ = 5.4 Hz), 8.38 (s, 2H, H5 and H6), 9.14 (dd, 2H, H4 and H7, *J*^3^ = 8.2 Hz, *J*^4^ = 1.1 Hz), 9.86 (dd, 2H, H2 and 9, *J*^3^ = 5.4 Hz, *J*^4^ = 1.1 Hz) ppm. ^13^C-NMR (DMF-d_7_) δ: 126.4 (C3 and C8), 128.2 (C5 and C6), 131.4 (C4a and C6a), 139.8 (C4 and C7), 148.4 (C1a and C10a), 149.4 (C2 and C9) ppm. ^195^Pt-NMR (DMF-d_7_) δ: −2322 ppm. Elemental analysis calcd. for C_12_H_8_Cl_2_N_2_Pt C, 32.30%; H, 1.81%; N, 6.28% found C, 32.51%; H, 1.76%; N, 6.20%.

### 4.3. Fluorescence Assays

ThT (50 µM, λ_exc: =_ 440 nm, λ_emis_ = 481 nm) fluorescence assays were carried out at 25 °C employing a peptide concentration of 100 µM in 10 mM phosphate buffer at pH = 7.4, at indicated ratios of Pt (II) complexes. To compare different effects of Pt complexes to peptides alone, correctly, analyzed solutions contained DMSO at 2% (*v*/*v*). Spectra were recorded with a Jasco FP 8300 spectrofluorometer (JASCO, Tokyo, Japan) in a 1cm cuvette under magnetic stirring. Spectra were recorded every 15 min in indicated time, assays were performed in duplicates, and reported intensities are averaged values.

### 4.4. UV/Vis Spectroscopy

UV/Vis titration of different Pt(II) complexes with Aβ_21–40_ were carried out employing a Nanodrop 200 c spectrophotometer (Thermo Scientific, Milan, Italy). To a fixed concentration of the Pt(II) complexes (20 µM), were added increasing amounts of Aβ_21–40_ of 2.0 µL of peptide stock solutions (500 µM) in water, kept at 0 °C. Ratios reached were 1:6 complex: Aβ_21–40_ for Pt-terpy and Pt-phen and 1:7.2 for Pt-bpy. Each spectrum was registered (220–400 nm) upon the addition of the peptide, after a stirring time of 2 min. EC_50_ value was derived from non-linear regression of the data employing log [inhibitor] vs. response and “dose-response stimulation equation” of GraphPad program [39].

### 4.5. ESI-MS Analysis

Solutions of Aβ_21–40_ at a concentration of 50 µM in 15 mM AMAC (Ammonium acetate) buffer pH = 7.0, at 1:5 ratios with Pt(II) complexes were incubated at two different times (0 and 24 h) and then analyzed by Q-ToF Premier (Waters, Milliford, MA, USA) mass spectrometer. The analyses were done by direct injection at 10 µL/min and the source parameters were set as 3 kV for capillary voltage and 42 kV for cone voltage. The acquisition range was spanned from 900 to 2500 *m*/*z* and the raw data were processed with MassLynx 4.1 software (Waters, Milliford, MA, USA). 

### 4.6. CD Spectroscopy

CD spectra of Aβ_21–40_ (100 µM, 10 mM phosphate buffer), with 1:1 and 1:5 ratios with Pt (II) terpy complex were registered at indicated times on a Jasco J-815 spectropolarimeter (JASCO, Tokyo, Japan), in a 0.1 cm cuvette. Deconvolutions of CD spectra were obtained by BESTSEL software (http://bestsel.elte.hu/, accessed on 20 February 2021) [40].

### 4.7. Cellular Assays

#### 4.7.1. Cell Proliferation Assay

Human neuroblastoma SH-SY5Y cell line was grown in DMEM (GIBCO, Paisley, UK) supplemented with 2 mM l-glutamine, 50 ng/mL streptomycin, 50 units/mL penicillin and 10% heat-inactivated fetal bovine serum (FBS) in a humidified atmosphere (5% CO_2_ at 37 °C). Cells were detached with 0.25% trypsin (Sigma-Aldrich, Milan, Italy) at 70–80% of confluence.

#### 4.7.2. Sulforhodamine B (SRB) Assay

To perform SRB assays, cells were seeded in triplicates in 96-well plates at a density of 8000 cells/well and left to adhere for 24 h. Aβ_21–40_ alone and Aβ_21–40_ + Pt-terpy at 1:5 ratio (after 0, 2, 24 h of stirring) were added to the culture in triplicates. After 24 h, cells were fixed with 50% *v*/*v* trichloroacetic acid (for 2 h at 4 °C) and then washed with water. Plates were dried overnight and then cells were stained with 0.4% *w*/*v* SRB in 1% *v*/*v* acetic acid at room temperature for 30 min on a shaker, and after washed with 1% acetic acid, to allow the removal the unbound dye [41]. They were resuspended with TRIS-HCl 10 mM, pH 7.4 and absorbance was measured at 495 nm employing a Glomax^®^ Discover Microplate Reader (Promega, Madison, WI, USA).

## Figures and Tables

**Table 1:** Peptide sequences analyzed in this study.

Peptide	Sequence
Aβ_21–40_	AEDVGSNKGAIIGLMVGGVV
mutAβ_21–40_	AEDVGSNKGAIIGLMVDGVV

**Table 2:** Summary of main ion species occurring in the ESI-MS spectra of Aβ_21–40_ with the three Pt-derivates, at 0 and 24 h of incubation. Each detected adduct is reported with the corresponding experimental *m*/*z* values, the charge state, the calculated and theoretical monoisotopic molecular weight.

Pt-Derivate	Time	Experimental *m*/*z*, (Charge State)	Experimental Monoisotopic Mass (Da)	Theoretical Monoisotopic Mass (Da)	Pt(II)-Peptide Complexes
Pt-phen	0 h	1150.52 (+2)	2299.06	2299.29	Aβ_21–40_ + 1 Pt-phen − 2HCl
		1180.04 (+2)	2358.07	2358.29	Aβ_21–40_ + 1 Pt-phen − 2HCl+AcO^−^
		1168.52 (+2)	2334.18	2335.74	Aβ_21–40_ + 1 Pt-phen − 1HCl
	24 h	1150.53 (+2)	2299.06	2299.29	Aβ_21–40_ + 1 Pt-phen −2HCl
		1180.04 (+2)	2358.07	2358.29	Aβ_21–40_ + 1 Pt-phen−2HCl+AcO^−^
		1168.50 (+2)	2335.00	2335.74	Aβ_21–40_ + 1 Pt- phen − 1HCl
Pt-bpy	0 h	1138.02 (+2)	2274.04	2275.27	Aβ_21–40_ + 1 Pt-bpy − 2HCl
		1168.05 (+2)	2234.10	2334.27	Aβ_21–40_ + 1 Pt-bpy − 2HCl+AcO^−^
		1156.50 (+2)	2311.00	2311.72	Aβ_21–40_ + 1 Pt-bpy − 1HCl
	24 h	1138.02 (+2)	2274.04	2275.27	Aβ_21–40_ + 1 Pt-bpy − 2HCl
		1156.50 (+2)	2310.00	2311.72	Aβ_21–40_ + 1 Pt-bpy − 1HCl
Pt-terpy	0 h	1176.56 (+2)	2351.14	2353.35	Aβ_21–40_ + 1 Pt-terpy − 1HCl
	24 h	1176.57 (+2)	2351.14	2353.35	Aβ_21–40_+ 1 Pt-terpy − 1HCl

## Data Availability

The data presented in this study are available on request from the corresponding author.

## Supplementary Materials

The following are available online at https://www.mdpi.com/1422-0067/22/6/3015/s1, Figure S1: ESI-MS spectra of mutAβ_21–40_ at 0 and 24 h of incubation in the absence and in the presence of Pt-terpy, Figure S2: Absorption spectra of Pt-phen, Pt-bpy and Pt-terpy upon the addition of increasing volume of solvents, Figure S3: Overlay of CD spectra of mutAβ_21–40_ in the absence and in the presence of Pt-terpy, Table S1: Deconvolution of CD spectra reported in Figure 5 at indicated times and ratios β_21–40_: Pt-terpy.
