# Supplementary material for: A Comparative Study of the Effects of Platinum (II) Complexes on β-Amyloid Aggregation: Potential Neurodrug Applications
Source: Int J Mol Sci. 2021 Mar 16;22(6):3015. doi: 10.3390/ijms22063015 (PMC7998721; doi:10.3390/ijms22063015)
Supplement: Supplementary file 1 [file ijms-22-03015-s001.pdf]

Supporting information:

Article

A Comparative Study of the Effects of Platinum (II) Complexes on β-Amyloid Aggregation: Potential Neurodrug Applications

Sara La Manna [1], Daniele Florio [2], Ilaria Iacobucci [3], Fabiana Napolitano [4], Ilaria De Benedictis [5], Anna Maria Malfitano [6], Maria Monti [7], Mauro Ravera [8], Elisabetta Gabano [9] and Daniela Marasco [10,*]

1. Department of Pharmacy, University of Naples "Federico II", 80131, Naples, Italy; sara.lamanna@unina.it
2. Department of Pharmacy, University of Naples "Federico II", 80131, Naples, Italy; floriodaniele1@gmail.com
3. Department of Chemical Sciences, CEINGE Biotecnologie Avanzate S.c.a r.l., "University of Naples Federico II", 80131, Naples, Italy; ilaria.iacobucci@unina.it
4. Department of Translational Medical Science, University of Naples "Federico II", 80131, Naples, Italy; fabiananapolitano94@gmail.com
5. Department of Pharmacy, University of Naples "Federico II", 80131, Naples, Italy; ilariadebenedictis2308@gmail.com
6. Department of Translational Medical Science, University of Naples "Federico II", 80131, Naples, Italy; annamaria.malfitano@unina.it
7. Department of Chemical Sciences, CEINGE Biotecnologie Avanzate S.c.a r.l., "University of Naples Federico II", 80131, Naples, Italy; montimar@unina.it
8. Department of Sciences and Technological Innovation (DiSIT), University of Piemonte Orientale "A. Avogadro", 15121, Alessandria, Italy; mauro.ravera@uniupo.it
9. Department of Sciences and Technological Innovation (DiSIT), University of Piemonte Orientale "A. Avogadro", 15121, Alessandria, Italy; elisabetta.gabano@uniupo.it
10. Department of Pharmacy, University of Naples "Federico II", 80131, Naples, Italy;

* Correspondence: daniela.marasco@unina.it; Tel.: (+39)081-2534512. Fax: (+39)081-2534574

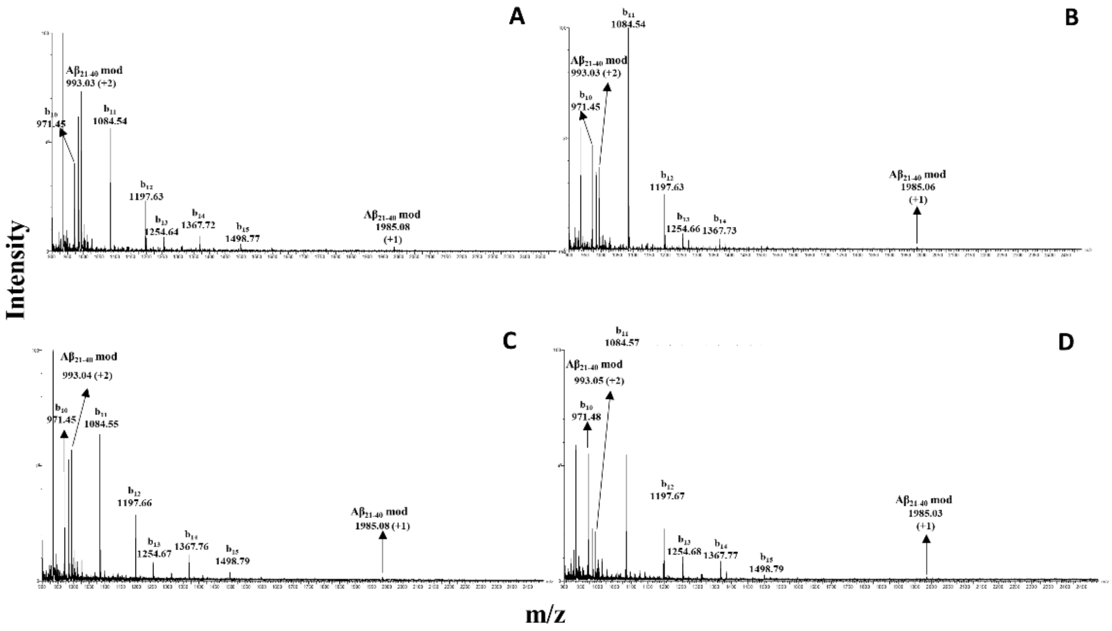

Figure S1. ESI-MS spectra of mutA$\beta_{21\text{-}40}$ at 0 and 24h of incubation in the absence (panel **A** and **B**, respectively) and in the presence of Pt-terpy (panel **C** and **D**, respectively). Fragmentation ions are also indicated.

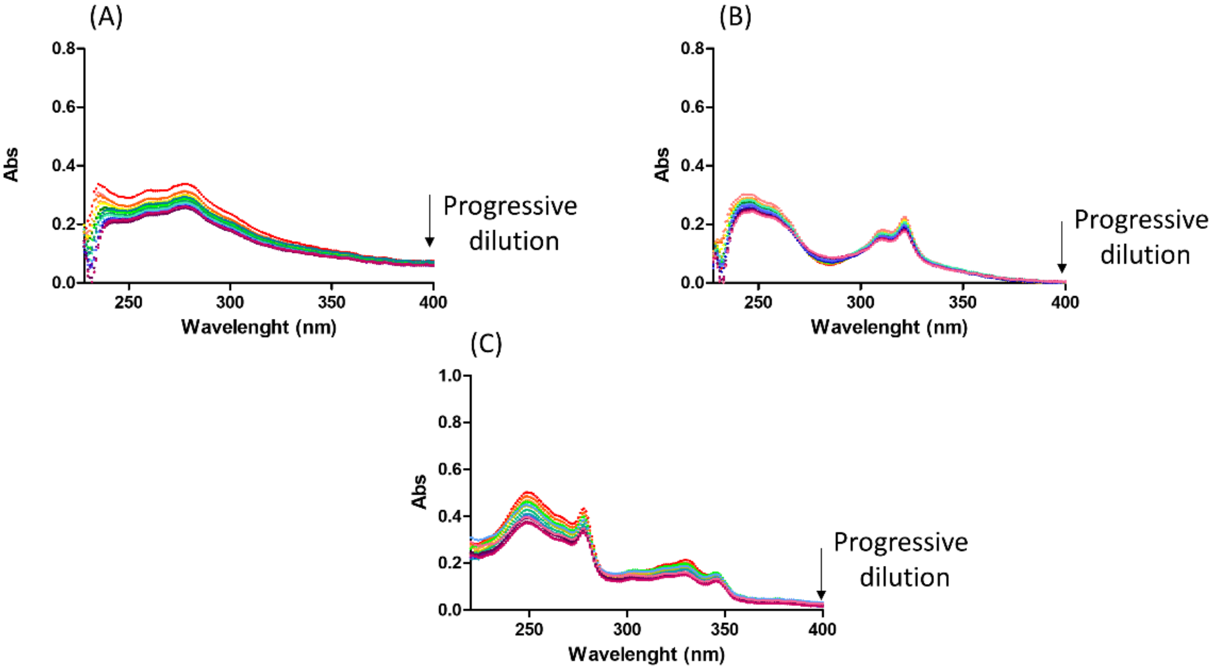

Figure S2. Absorption spectra of (**A**) Pt-phen, (**B**) Pt-bpy, (**C**) Pt-terpy upon the addition of increasing volume of solvents Arrow indicates increasing dilution.

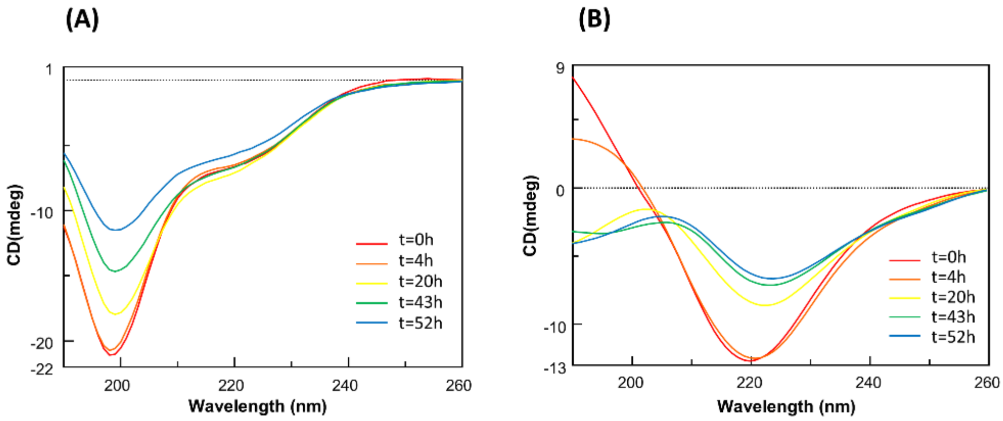

Figure S3. Overlay of CD spectra of mutAβ_{21-40} (**A**) alone, (**B**) with Pt-terpy complex at 1:5 peptide: Pt(II) compound molar ratio.

Table S1. Deconvolution of CD spectra reported in Figure 5 at indicated times and ratios β_{21-40}: Pt-terpy.

	Time (h)	Helix%	Beta %	Turn%	Random coil%
Aβ_{21-40}	0	2.8	23.4	18.7	55.0
Aβ_{21-40}: Pt-terpy 1:1	0	15.1	34.1	5.9	44.9
Aβ_{21-40}: Pt-terpy 1:5	0	19.7	37.3	3.5	39.6
